# Evaluating appropriateness and clinical impact of a GI PCR panel: A Retrospective Study

**DOI:** 10.1017/ash.2025.313

**Published:** 2025-09-24

**Authors:** Pranav Ramamurthy, Jennifer Schimmel, Sherin Samuel, Elisha Shah, Kevin Groudan, Lydia D’Agostino, Yesenia Greeff

**Affiliations:** 1 UMass Chan - Baystate Medical Center; 2 Baystate Health; 3 University of Massachusetts Chan Medical School - Baystate; 4 UMass Chan -Baystate; 5 University of Massachusetts Chan-Baystate

## Abstract

**Background:** Acute gastroenteritis and diarrheal illnesses have a significant burden on the United States healthcare system, with over 500,000 estimated hospitalizations annually. Testing for these conditions is often ordered inappropriately at significant cost to the healthcare system. This study aimed to determine the appropriateness of ordering of gastrointestinal PCR panel (GIP) testing in our hospital system to guide improvements in ordering practices. It also aimed to evaluate the impact of a GIP in our system. **Method:** This was a retrospective chart review with the objective of quality improvement. The appropriate measures for ordering a GIP test included documentation of diarrhea in addition to fever, blood in stool, signs of sepsis or immunocompromise and without history of laxative use in preceding 48 hours. The result of a positive versus negative GIP test was measured in terms of its effect on isolation time and appropriate de-escalation of antibiotics. **Result:** Of the 402 records which were reviewed, 204 (50.7%) were deemed to have had an appropriately ordered test per our criteria. However, of these patients, 21 were noted to have either been on tube feeds or had received bowel regimen medications within the past 48 hours. When these patients were excluded, this left 183 (45.5%) patients with an appropriately ordered GIP test. Of note, 16 of these patients had a positive concomitant C. difficile test. Of the 93 (23.1%) positive tests, only 36 positive results were from appropriately ordered tests of which 9 tests impacted clinical management. Of the 57 remaining tests, 11 impacted clinical management. A negative test led to discontinuation of isolation precautions in 159 (76.1%) patients who had isolation placed for diarrheal illness prior to testing. Negative tests also led to discontinuation of antibiotics in 51 (39.5%) patients. There was no difference between these groups regardless of whether the test was ordered appropriately or not. **Conclusion:** The GIP test to detect a variety of gastrointestinal pathogens is not being ordered appropriately in our health system over half the time. It bears further investigation as to whether the monetary cost to patients and the health system of this test is offset by the apparent antibiotic stewardship and cost benefits in discontinuing isolation precautions and antibiotics. Interestingly, testing appeared to have utility regardless of appropriateness. Based on this finding, an updated set of guidelines to educate physicians in the appropriate ordering and interpretation of this test is required.

